# Numerical Simulation of Solid Combustion in Microporous Particles

**DOI:** 10.3389/fchem.2020.510686

**Published:** 2020-10-07

**Authors:** Gérald Debenest, Romain Guibert, Pierre Horgue, Chen Yang

**Affiliations:** ^1^Institut de Mécanique des Fluides de Toulouse (IMFT) - Université de Toulouse, CNRS-INPT-UPS, Toulouse, France; ^2^College of Chemical Engineering, Fuzhou University, Fuzhou, China

**Keywords:** smoldering, direct numerical simulations, Darcy-Brinkman model, solid combustion, heat and mass transfer

## Abstract

In the present study, direct numerical simulations for smoldering in simplified geometries are performed for multicomponent and dilatable flows. The reactant gas is passing inside an array of permeable and microporous cylinders. The chemical reaction takes place within the grains. For the sake of simplicity, the smoldering is treated as a single step chemical reaction. We define a Darcy-Brinkman model to deal with the multi-scale nature of this problem. Varying the flow rate, and carbon content, we can investigate the response of our model, and try to compare with existing solutions or available experimental investigations. Thus, the ability of the Darcy Brinkman approach to capture such situations of porous particles combustion will be checked. The numerical model sensitivity to the inner grain permeability is also investigated.

## 1. Introduction

Smoldering consists in a heterogeneous flameless combustion. The fuel could either be a liquid or a solid residue which is oxidized by a reactant. This process is slow and sustained by the heat generated from the oxidation reaction. This is a quite well-known process at the pore scale and all the possible regimes have now be exhibited using two different approaches:

– An incompressible approach, i.e., with no dependence of the diffusive flux from the other gas phase concentrations (Debenest et al., 2005a,b, 2008);
– A dilatable approach (Yang and Debenest, 2014), which means that the density varies with the composition and the temperature.

Those studies have demonstrated that the analytical solutions proposed by Dosanjh et al. (1987), based on simplified assumptions, do not change due to the compositional effects or dilatation. Aldushin et al. (1976) have described this complex phenomenon by conducting an one-dimensional theoretical analysis of combustion propagation wave in a porous media. Using an asymptotic method, the structure of the solution has been determined and can be summarized depending on three different cases:

– If the heat velocity front is slower than the reaction front, a *reaction leading* wave structure appears;
– If the heat velocity is quicker than the reaction front, the so-called *reaction trailing* regime appears (Wahle and Matkowsky, 2001);
– Finally, if the two traveling waves move at the same speed, the superadiabatic regime appears leading to an infinite amount of energy in the reaction region (Aldushin et al., 1999).

Akkutlu and Yortsos (2003) studied *in-situ* in order to derive an new analytical in order to determine concentrations, temperature but also front velocity when heat losses are taken into account.

**Figure 1:**
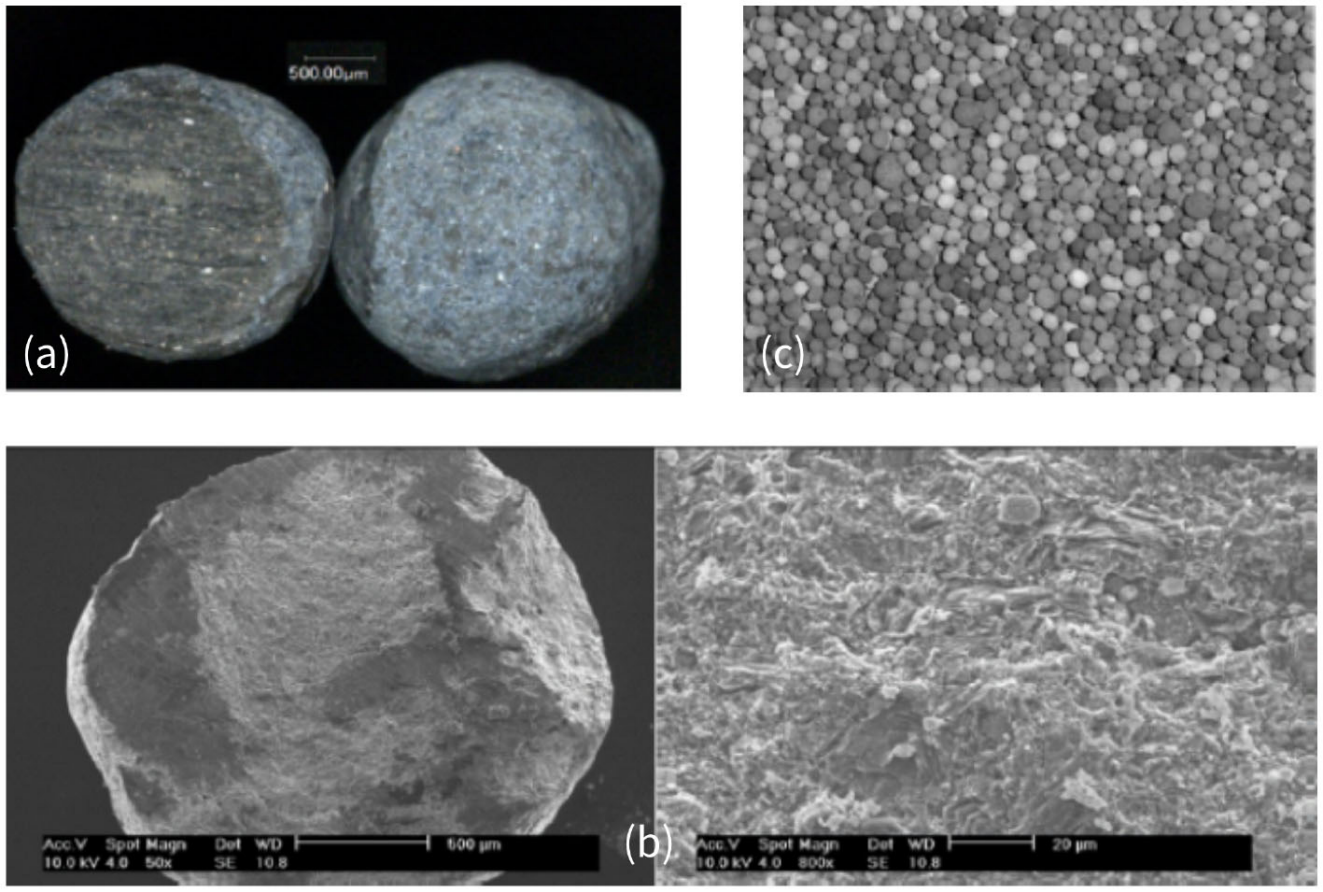
**(a)** Optical microscope view of particles filled with carbon: (left) cut-away and (right) external view. **(b)** SEM cut-away view of carbonaceous particles filled with carbon at a magnification of 50 and 800. **(c)** Photograph of a bed of particles (picture actual width: 5 cm). Picture of the porous bed made with alumina microporous spheres. Reprinted (adapted) with permission from Baud et al. (2015). Copyright (2015) American Chemical Society.

In several recent papers, those regimes were studied both numerically (Yang and Debenest, 2014; Yang et al., 2015b) and experimentally (Sennoune et al., 2011; Baud et al., 2015), which allowed a better characterization of the reaction behavior depending on operating conditions. By varying the reactive content, the oxygen concentration, or material properties (density for instance), one could change the wave dynamics. Baud et al. (2015) have studied in details the behavior of the combustion front using a new synthetic porous material, mimicking bituminous sands. This porous medium, illustrated in Figure 1, consists of microporous spheres containing reactive carbon. These observations revealed that, at the scale of a particle, the repartition of the carbon is homogeneous (Figure 1a). The color within the particle is uniform. The scanning electron microscope photographs in Figure 1b confirmed very small pore sizes inside the medium. At the scale of the bed, we observe possible inhomogeneity in the carbon content as reported in Baud et al. (2015). However, the particles have a small size compared to the chemical front thickness in standard conditions and the particles are well-mixed. So, even if inhomogeneity of carbon contents exists, it is still reasonable to assume homogeneous medium on the front thickness scale.

By choosing different impregnation times for alumina microporous spheres, Baud et al. (2015) demonstrated that the amount of carbon could be controlled. Then, this synthetic medium is used to study the behavior of the reactive zone as the heat wave, and compared then with theoretical results. A major observation was that an incomplete combustion in the solid matrix could lead to a large increase of temperature. Even if this was expected from the analytical solution, this experimental carbon front characterization allows a better understanding of the couplings between reaction rate, heat and mass transfers around and within the grains. The modeling of this multi-scale processes usually consists in macro-scale approach where the porous matrix and the gas phase are described via a Darcy-scale model and where the microstructure effects are embedded in effective macro-scale properties (Fadaei et al., 2012). Pore scale approaches have been also investigated in 2 and 3D packing of spheres in order to exhibit local scale effects, like thermal non-local equilibrium or inert material effects for instance (Debenest et al., 2005b). Some other approaches are using pore network models, where they rely on the representation of the void space within porous materials as a network of interconnected pores with idealized geometries. In Lu and Yortsos (2004), they used a randomness distribution of reactive material to investigate the propagation of the smoldering process emphasizing the effects of heat losses, fuel location and availability. In Lu and Yortsos (2005), the case of reverse combustion using the same pore network approach is studied. due to heat losses effects, and depending on the Péclet, they found unstable propagation.The numbers of fingers, their sizes depended on the flow rate but also on the transport parameters.

In the case studied in Baud et al. (2015), the medium is complex and two scales coexist. Phenomena are complex, including:

– In the gas phase, transfers are modified by the presence of solid microporous particles;
– In the particles, the micro-pores are partially filled with reactive carbon;
– Transfers and reactions occur in both phases (fluid and porous matrix).

In order to describe this complex medium and its multi-scale flows we choose to use a Darcy-Brinkman model (DBM) (Brinkman, 1949). Navier-Stokes equations are solved within the gas phase, and a Darcy equation is used for the microporous phase i.e., in the particles. This approach allows to couple several scales by adding a coupling term depending on the porosity and the permeability within the porous domain. This DBM is coupled with multicomponent transport equation for all of the main components conveyed in the gas phase. The reaction is modeled using an one step kinetic inside the porous particles where an effective reactivity is defined.

In this study, smoldering in simplified geometries is considered. We use several idealized geometries (one-dimensional configuration and arrays of two-dimensional cylinders) to investigate the influence of parameters like carbon content, flow rate and temperature levels. Firstly, we will describe the geometry and the model with its main assumptions. A complete set of governing equations for dilatable flow will be given according to the description of Yang and Debenest (2014). After the validation step, the numerical study is compared with available results (Baud et al., 2015). Then, illustrating two-dimensional geometries are considered to demonstrate the ability of this class of model to represent more complex situations (variations of effective properties, carbon content concentration, etc.).

## 2. Materials and Methods

**Figure 2:**
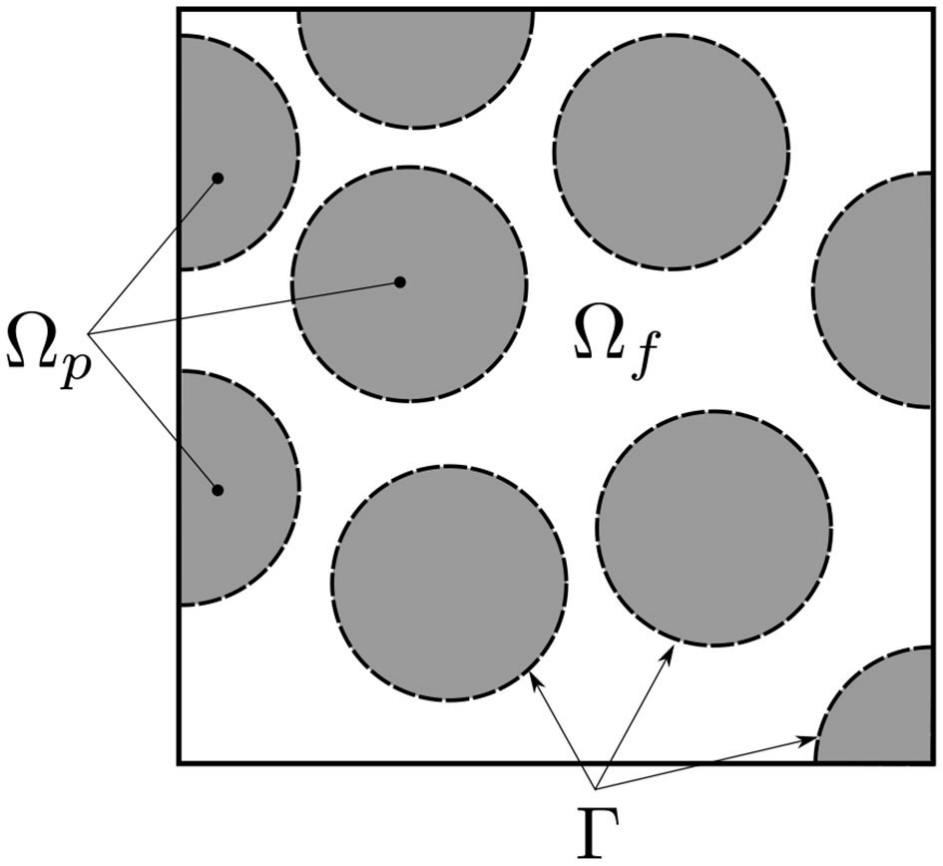
Definition of the different domain regions: Ω = Ω_*f*_ ∪ Ω_*p*_ and Γ the interface between Ω_*f*_ and Ω_*p*_.

As illustrated in Figures 1, 2, the gas is flowing through a porous medium which consists of an array of cylinders. The gray area is the matrix where the void space is partially filled with carbon deposits. The gaseous species flow around spheres, but can also diffuse inside the porous material and react with the immobile carbon.

The smoldering process, which is simplified in a single step chemical reaction, takes place in the matrix volume. The considered reaction corresponds to an homogeneous system rather than an heterogeneous reactive one as those previously studied in Debenest et al. (2005b). This has been previously stated in several studies (Ohlemiller, 1985; Torero and Fernandez-Pello, 1996; Martins et al., 2010). This is a strong assumption in terms of chemical model, because the chemistry is much more complex with hundreds of steps and components. In the case of carbon oxidation, which is a comparable situation, the general chemical scheme usually provides a three or four steps scheme. One can find a summary in Elayeb et al. (2007). But, the combustion of a carbon residue is often treated using a four effective reactions scheme like the one determined in Vzajdik et al. (2001). In this work, we introduce a carbon monoxide fraction *fr*_*CO*_ representing the partial oxidation of the carbon residue into carbon mixed *CO* and carbon dioxide *CO*_2_ (Pozzobon et al., 2016). It is important to note that our situation is not comparable with a complete pore-scale study because the porous medium is modeled as an effective one. Thus, the reaction is homogeneous and depends on temperature, carbon content, and oxygen concentration. To investigate this complex situation, different geometries will be used and detailed further.

### 2.1. Mathematical Model

The proposed model is based on a complete description of transports within the considered porous medium. Momentum, transport and heat transfer equations are derived from the model developed in Yang and Debenest (2014). The domain definition is illustrated in Figure 2.

#### 2.1.1. Hydrodynamics

Considering a compressible newtonian fluid, the continuity equation writes

(1) $$\begin{array}{l} \frac{\partial \rho }{\partial t}+\nabla .\left(\rho \text{u}\right)=0 \end{array}$$

where ρ is the density and **u** the velocity. The momentum equation coupling free fluid flow and porous media flow is given by

(2) $$\frac{\partial \rho \text{u}}{\partial t}+\nabla \cdot (\rho \text{>uu})=-\nabla (p+\frac{2}{3}\mu \nabla \cdot \text{u})-\frac{\mu_{e}}{\text{K}}\text{u}+\nabla \cdot \left[\mu (\nabla \text{u}+{\left(\nabla \text{u}\right)}^{T}\right]$$

where *p* is the pressure, μ the viscosity in the fluid domain, μ_*e*_ the viscosity in the porous medium and **K** the permeability tensor. In the studied cases, porous medium is considered as isotropic and characterized by a scalar permeability. The penalization term $\frac{\mu_{e}}{\text{K}}\text{u}$ permits to deal with an unique domain solving a Darcy equation in the porous media Ω_*p*_ and Navier-Stokes equations in the free region Ω_*f*_. At the interface Γ between the two regions, the continuity of pressure and flux are implicitly imposed. In the literature, Brinkman (1949) and Haber and Mauri (1983) assumed the viscosity in all regions to be equal. This is not a general case as stated in Givler and Altobelli (1994) where μ_*e*_ is larger than the fluid viscosity. For the sake of simplicity , we will assume this equality. We have imposed the porosity and permeability values to be respectively 0.6 and 10^−14^ m^2^. The sensitivity of the model to the permeability values will be checked in the following. This continuum DBM model is an appropriate approach for the complex situations under investigation in this work.

#### 2.1.2. Transports and Reactions

Regarding the species transport within the medium, a classical convection/diffusion equation with a source term is used. In the free region Ω_*f*_, there is no reaction and the transport follows

(3) $$\begin{array}{l} \frac{\partial \rho Y_{f,i}}{\partial t}+\nabla {\cdot \text{J}}_{f,i}=0 \end{array}$$

where *Y*_*f,i*_ is the mass fraction of species *i* and **J**_*f,i*_ is the total mass flux defined by

(4) $$\begin{array}{l} {\text{J}}_{f,i}=\rho \left(\text{u}Y_{f,i}-D_{f,i}\nabla Y_{f,i}\right) \end{array}$$

with **D**_*f,i*_ the molecular diffusion of species *i*. In the porous region Ω_*p*_, the transport equation is modified due to the restriction related to the porous region porosity ε_*p*_. In Ω_*p*_, the advection/diffusion equation becomes

(5) $$\frac{\varepsilon_{p}\partial \rho Y_{p,i}}{\partial t}+\nabla \cdot {\text{J}}_{p,i}=R_{i}$$

where *R*_*i*_ are the reactive terms related to species *i*, with the total mass flux

(6) $$\begin{array}{l} {\text{J}}_{p,i}=\rho \left(\text{u}Y_{p,i}-\varepsilon_{p}{\text{D}}_{p,i}^{e}\nabla Y_{p,i}\right) \end{array}$$

where ${\text{D}}_{p,i}^{e}$ stands for the effective dispersion, accounting for mechanical diffusion due to the micro-pore but also to dispersion effect, due to the velocity field variations within the medium. The dispersion effect generally occurs when local Péclet Number values are larger than 10. In Baud et al. (2015), the porous domain consists of alumina particles with a diameter *L*_*ref*_ equals to 2.5 mm. In this work, the particles are microporous with a measured mean inner diameter, Φ close to 40 nm. With standard values of the diffusion coefficients (~ 10^−5^ m^2^/s) and using reference values of the velocity within the porous particles, one can estimate a particle Péclet number *Pe*_*p*_ and an intra-granular particle Péclet number *Pe*_*in*_ following

(7) $$\begin{array}{l} Pe_{p,i}=\frac{V^{*}L_{ref}}{D_{i}}, \end{array}$$

with *i* = *O*_2_, *N*_2_, *CO*_2_, *CO* and

(8) $$\begin{array}{l} Pe_{in}=\frac{V_{in}\Phi }{D_{i}} \end{array}$$

where the interstitial velocity is defined by $V^{*}=\text{u}/\varepsilon_{p}$ and *V*_*in*_ is the intra-granular particle velocity. According to Baud et al. (2015), standard values of velocity range from 0.02 to 0.2 m/s around the particles, and could be with several orders of magnitude less within the grains. Thus, we could estimate Péclet, *Pe*_*in*_ ≪ 1. In this conditions, the effective diffusion reduces to the mechanical diffusion. Thereby, the effective diffusion is

$${\text{D}}_{p,i}^{e}=\frac{D_{i}}{\tau }\text{I}$$

where *D*_*i*_ is the molecular diffusion and τ is the tortuosity of the porous medium, always higher than unity.

Several studies have proposed a measurement of the tortuosity (Wang and Smith, 1983; Barrande et al., 2007) using different techniques on different samples. In Wang and Smith (1983), the catalyst pellets packing studied which are close to the ones used in Baud et al. (2015), presented a tortuosity of 8 that we choose as our reference.

All the species are considered as diluted and to enforce the mass fractions conservation the diazote *N*_2_, which is the diluent, obeys the following relation

(9) $$\begin{array}{l} Y_{N_{2}}=1-\underset{i\neq N_{2}}{\sum }Y_{i}. \end{array}$$

Note that the effects of multicomponent transports is neglected and this study focuses on the coupling between transport phenomena in a multi-phase system. Moreover, the diffusion coefficients are supposed constant without the considerations of concentration and temperature variations. The conservation of solid the reactive specie, the carbon, writes

(10) $$\begin{array}{l} \frac{\partial \rho_{C}}{\partial t}=-R_{C} \end{array}$$

with ρ_*C*_ is the mass concentration of carbon and *R*_*C*_ is the reaction rate of carbon in the porous region that is described by the following Arrhenius law

(11) $$\begin{array}{l} R_{C}=Aexp\left(-\frac{E_{a}}{RT}\right)\rho_{C}Y_{O_{2}} \end{array}$$

with *A* the pre-exponential factor, *E*_*a*_ the activation energy, *R* the universal gas constant and *T* the temperature. The values of constants *A* and *E*_*a*_ are those determined in Baud et al. (2015) using ATG experiments:

$$A=0.327{\text{ s}}^{-1}\text{ and }E_{a}=18500\text{ J/mol.}$$

One can note that the low activation energy confirms that a mass transfer limitation regime exists.

The smoldering of solid carbon with oxygen is underlain by complex chemical and thermal mechanics featuring both heterogeneous and homogeneous reactions taking place within the particles. After combustion, part of the carbon will end up as CO and part as CO_2_. A global description of the oxidation is synthesized by

(12) $$\begin{array}{l} C+\left(1-fr_{CO}/2\right)O_{2}\to fr_{CO}CO+\left(1-fr_{CO}\right)CO_{2} \end{array}$$

The parameter *fr*_*CO*_ is affected by various parameters such as air flow rate, temperature, carbon content and geometrical configuration. This parameter strongly affects the smoldering process because it impacts the chemical front velocity which varies by a ratio of 2 when *fr*_*CO*_ changes from 0 to 1 and the released energy by carbon combustion Δ*H* changes from 393.5 to 110.5 kJ/mol when *fr*_*CO*_ changes from 0 to 1. These two points result in a drastic change in the front temperature.

Moreover, this parameter is difficult to assess. Experimental results available mainly concern single particles (Manor et al., 1982; Tognotti et al., 1991) for various settings, different ranges of temperature and different oxygen concentration. However, use one of these *fr*_*CO*_ values remains questionable when modeling combustion of a packed bed. Closer to the studied configuration, Sennoune et al. (2011) have measured this ratio depending on the carbon content and a numerical model has been proposed (Klimenko and Abdel-Jawad, 2007) to recover the values of *fr*_*CO*_. In all of these cases, *fr*_*CO*_ remains a value dependent on local thermochemical conditions, the composition, boundary conditions and bed configuration.

In this study, we take an average value of 0.3, not depending on any operating conditions, as determined in Baud et al. (2015). The reaction rate is a first order kinetic with respect to oxygen concentration and carbon content. Finally the reaction rates for the species involved are:

(13) $$\begin{array}{l} R_{O_{2}}=-\left(1-fr_{CO}\right)R_{C}\frac{M_{O_{2}}}{M_{C}}-fr_{CO}R_{C}\frac{M_{O_{2}}}{2M_{C}}, \end{array}$$

(14) $$\begin{array}{l} R_{CO_{2}}=\left(1-fr_{CO}\right)R_{C}\frac{M_{CO_{2}}}{M_{C}}, \end{array}$$

(15) $$\begin{array}{l} R_{CO}=fr_{CO}R_{C}\frac{M_{CO}}{M_{C}}. \end{array}$$

#### 2.1.3. Heat Transfers

In the free fluid region Ω_*f*_, the heat transport follows

(16) $$\begin{array}{l} \frac{\partial {\left(\rho C_{p}\right)}_{f}T_{f}}{\partial t}+\nabla {\cdot \text{J}}_{f}^{T}=0 \end{array}$$

where the thermal flux ${\text{J}}_{f}^{T}$ is defined by

(17) $$\begin{array}{l} {\text{J}}_{f}^{T}={\left(\rho C_{p}\right)}_{f}\text{u}T_{f}-\lambda_{f}\nabla T_{f} \end{array}$$

with ${\left(\rho C_{p}\right)}_{f}$ the heat capacity of the free fluid region and λ_*f*_ the conductivity.

In the porous region, these equations are modified due to the presence of solid and gaseous phases. The particles are considered under a local thermal equilibrium, so a single temperature model is assumed within the grains. Oliveira and Kaviany (2001) have identified the length and time scales involved in heat and mass transports during combustion in porous media and have determined the conditions for the use of a local equilibrium assumption. Both high Péclet and Damköhler values are the conditions leading to the development of more elaborate thermal non-equilibrium models. As stated in Yang et al. (2015b) and Debenest et al. (2005b), local thermal equilibrium is enhanced when increasing both Péclet and Damköhler values which has also been observed using local simulations with heterogeneous reactions. A special Péclet number has been specially designed to exhibit the possible non-equilibrium effects. It is defined as follow:

(18) $$\begin{array}{l} Pe_{F,s}=\frac{V_{f}L_{ref}}{D_{s,th}} \end{array}$$

with $D_{s,th}=\frac{\lambda_{p}^{e}}{{\left(\rho C_{p}\right)}_{p}^{e}}$, the thermal diffusivity of the solid phase. The term $\lambda_{p}^{e}$ is the effective conductivity and ${\left(\rho C_{p}\right)}_{p}^{e}$ is the effective heat capacity and is defined as:

(19) $$\begin{array}{l} {\left(\rho C_{p}\right)}_{p}^{e}=\left(1-\varepsilon_{p}\right){\left(\rho C_{p}\right)}_{s}+\varepsilon_{p}{\left(\rho C_{p}\right)}_{f} \end{array}$$

with ε_*p*_ the micro-porosity of particles and ${\left(\rho C_{p}\right)}_{s}$ the heat capacity of solid particles. As previously seen, Péclet values remain low in the grains, for mass and thermal transport. Then, the classical one equation model for heat transport equation is used

(20) $$\begin{array}{l} \frac{\partial {\left(\rho C_{p}\right)}_{p}^{e}T_{p}}{\partial t}+\nabla \cdot {\text{J}}_{p}^{T}=Q \end{array}$$

with $C_{p}^{e}$ the effective heat capacity within particles. The source term *Q* and the flux ${\text{J}}_{p}^{T}$ are defined by

(21) $$\begin{array}{l} Q=R_{C}\Delta H_{r}, \end{array}$$

(22) $$\begin{array}{l} {\text{J}}_{p}^{T}={\left(\rho C_{p}\right)}_{p}^{e}\text{u}T_{p}-\lambda_{p}^{e}\nabla T_{p}, \end{array}$$

All the heat capacities are dependent both on the composition and the temperature but we assume in this study a constant average value of heat capacity.

Such as in the mass transport equation, the heat transport is also affected by the flow with a dispersive contribution. The thermal Péclet values remain always low, so the dispersivity will only depend on the microstructure. This is well-studied in the literature (Nozad et al., 1985; Quintard et al., 1997; Yang et al., 2015a). Effect of heat sources, contact points and also the discussion on the use of periodic geometries are addressed within these references.

In our case, the particle conductivity has been measured and is given by the manufacturers. This value is close to 0.2 W/m/K (Baud et al., 2015). This value is used without any corrections in order to take into account the radiative heat transfers inside the grains. This might be of importance, but for simplicity no dependence of this conductivity with the heat transport phenomenon is considered, even if those effects could be significant between particles. The treatment of these transfers would require a complete study and is beyond the scope of this paper. Anyway, the reader can refer to the work of Yang et al. (2015b) where an Appendix explains the possibility to not treat this in the case of small pores.

The gas variations of density and dynamic viscosity are described by:

(23) $$\begin{array}{l} \rho =\frac{p}{RT\sum_{i}\left(Y_{i}/M_{i}\right)} \end{array}$$

(24) $$\begin{array}{l} \mu =\underset{i}{\sum }X_{i}\mu_{i} \end{array}$$

where *M*_*i*_ and *X*_*i*_ are respectively the molar mass and the molar fraction of each species *i*. In addition, the dynamic viscosity of pure gas can be calculated using,

(25) $$\begin{array}{l} \mu_{i}=\frac{AT^{B}}{1+C/T+D/T^{2}} \end{array}$$

with the coefficients proposed by Chapman and Cowling (1970), and reported in Table 1.

**Table 1:** Coefficients for the dynamic viscosity determination using Equation (25) of the different species involved in the studied process.

	**A**	**B**	**C**	**D**
*O*_2_	1.1 × 10^−6^	0.56	96.3	0
*CO*	1.8 × 10^−6^	0.5	140	0
*CO*_2_	2.14 × 10^−6^	0.46	290	0
*N*_2_	6.56 × 10^−7^	0.61	54.7	0

#### 2.1.4. Boundary Conditions

The boundary conditions for compressible flow are set as follows:

– At the inlet: the velocity is fixed, the concentration are fixed (**u** = **u**_*in*_, *Y*_*O*_2__ = 0.2, *Y*_*CO*_2__ = *Y*_*CO*_ = 0 and *Y*_*N*_2__ = 1−*Y*_*O*_2__) and the temperature is fixed (*T*_*f*_ = 650*K*)
– At the outlet, a free convective flux condition is imposed for all transport equations, and a pressure condition is imposed,
– On the lateral sections, a symmetry boundary condition is applied (no flux) for all quantities,
– At the interface: continuity of pressure, concentrations, temperature and velocity.

The parameter values used for the simulations are the same as that of Baud et al. (2015) and are reported in Table 2.

**Table 2:** Numerical values used for parameters.

**Parameters**	**Values (units)**
${\left(C_{p}\right)}_{s}$	1,000 J/kg/K
${\left(C_{p}\right)}_{f}$	1,145 J/kg/K
λ_*f*_	0.02 W/m/K
$\lambda_{p}^{e}$	0.2 W/m/K
ε_*p*_	0.61

### 2.2. Studied Geometries

**Figure 3:**
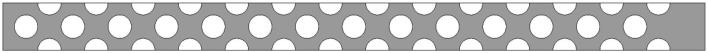
Sketch of the symmetric permeable array of cylinders. The length scales used are Φ = 2.5 mm for each particle diameter and *H* = 2Φ for the geometry height. The porosity is 0.633.

Three different geometries are used for this study. Firstly, an one-dimensional macro-scale configuration is studied in order to validate the model by comparison with the expected analytical predictions (Debenest et al., 2005a; Yang and Debenest, 2014). Then, a symmetric array of permeable cylinders is used. The domain is illustrated on Figure 3, which corresponds to a simplified porous region with porous particles (where the Darcy's equation and transports are defined) and a free region (in which Navier-Stokes and transport equations are used).

**Figure 4:**
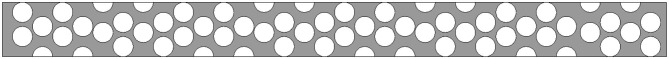
Sketch of the mono-disperse porous media used for the comparisons with available experimental data. Typical length scales used are Φ = 2.5 mm for each particle diameter and *H* = 2.7Φ for the geometry height. The porosity is 0.506.

Figure 4 represents a variant of a mono-disperse array of cylinders, that is used to compare numerical simulations with the experiments.

These two-dimensional media are characterized by two porosities:

– The macro-porosity ε defined by the ratio between the volume of free fluid region and the whole domain volume ($\varepsilon =\frac{V_{f}}{V_{f}+V_{p}}$, with respectively *V*_*f*_, *V*_*p*_, the volume of the free fluid region and the volume of porous particles);
– The micro-porosity within the particles, denoted ε_*p*_, corresponding to the void space available within the particles divided by the volume of the particles. As reported in Baud et al. (2015), this value is close to 0.6. As a matter of fact, it will modify the estimate of intra-granular heat capacity, conductivity, and dispersivity.

It is important to remind that Baud et al. (2015) measured the porosity by weighing a metered volume of the particle bed. According to the density value of the packed bed (of 808 kg/m^3^), this represents a porosity close to 0.45. In the case of the second geometry, we are close to the values of experimental observations.

### 2.3. Analytical Solutions and Governing Parameters

Interesting informations can be obtained from the simple situation for which the smoldering process is mono-dimensional and quasi-steady. This configuration permits to exhibit analytical solutions and determine predominant governing parameters.

We consider a macroscopic domain containing two phases (gas and solid) and characterized by porosity ε. The front is moving at a constant speed, *U*_*F*_ as oxygen consumes the reactive solid residue. At each time, the front is located at *x*_*F*_. The simplest description of this problem is a heat transport equation with a mobile heat source located at *x*_*F*_ coupled with a mass transport equation for oxygen and a sink term for the reaction (Dosanjh et al., 1987). The only necessary values are the concentration of oxidant and carbon residue at the inlet and outlet. Then, the molar quantity *C*_*O*_2_,*in*_ − *C*_*O*_2_,*out*_ reacts at *x*_*F*_. The initial carbon molar concentration deposited in the medium is initialized with *C*_*C,in*_ and the carbon unburnt after the passage of the front is *C*_*C,out*_. The front velocity *U*_*F*_ is estimated using the concentration balance,

(26) $$\langle \text{u}\rangle (C_{O_{2},in}-C_{O_{2},out})=(1-\varepsilon )U_{F}(C_{C,out}-C_{C,in})$$

with **〈*u*〉** the average gas velocity. In order to proceed a little bit further, let us introduce two simplifying hypotheses which are classically used in macroscopic models (Schult et al., 1996). Assume that gas and solid phases are in local thermal equilibrium, so that a description in terms of a single (position dependent) temperature is possible, and that the reaction zone is narrow. The heat transport equation, in a referential attached to the reaction front located at *x*_*F*_, takes the following form

(27) $${\left(\rho C_{p}\right)}^{e}\frac{\partial T}{\partial t}+\left({\left(\rho C_{p}\right)}_{g}\langle \text{u}\rangle -{\left(\rho C_{p}\right)}^{e}U_{F}\right)\frac{\partial T}{\partial x}-{\text{λ}}^{e}\frac{\partial^{2}T}{\partial x^{2}}=\delta_{x_{F}}S_{H}$$

with ${\left(\rho C_{p}\right)}^{e}$ the effective heat capacity, ${\left(\rho C_{p}\right)}_{g}$ the gas phase heat capacity, λ^*e*^ the effective thermal conductivity and *S*_*H*_ the source term located at the front. Like previously seen, using simple balance equations, this source term can rely on the front velocity using the relation

(28) $$\begin{array}{l} S_{H}=\left(1-\varepsilon \right)\left(C_{C,in}-C_{C,out}\right)U_{F}△H_{r} \end{array}$$

with △*H*_*r*_ the reaction enthalpy. According to this relation, one can define the adiabatic temperature *T*_*ad*_ which is the ratio of the produced heat by the reaction to the global heat capacity:

(29) $$\begin{array}{l} T_{ad}=\frac{\left(1-\varepsilon \right)\left(C_{C,in}-C_{C,out}\right)△H_{r}}{{\left(\rho C_{p}\right)}^{e}}=\frac{S_{H}}{U_{F}{\left(\rho C_{p}\right)}^{e}}. \end{array}$$

Let us define the effective thermal diffusivity

(30) $$\begin{array}{l} D^{e}=\lambda^{e}/{\left(\rho C_{p}\right)}^{e}. \end{array}$$

One can rewrite the Equation (27) as

(31) $$\frac{\partial T}{\partial t}+(\frac{{\left(\rho C_{p}\right)}_{g}}{{\left(\rho C_{p}\right)}^{e}}\langle \text{u}\rangle -U_{F})\frac{\partial T}{\partial x}-D^{e}\frac{\partial^{2}T}{\partial x^{2}}=\delta_{x_{F}}U_{F}T_{ad}$$

This equation is easy to solve and the solution is trivial. One can define an equivalent length,

(32) $$\begin{array}{l} L=\frac{D^{e}}{U_{F}|1-△|} \end{array}$$

with

(33) $$\begin{array}{l} \Delta =\frac{{\left(\rho C_{p}\right)}_{g}\langle \text{u}\rangle }{{\left(\rho C_{p}\right)}^{e}U_{F}}. \end{array}$$

**Figure 5:**
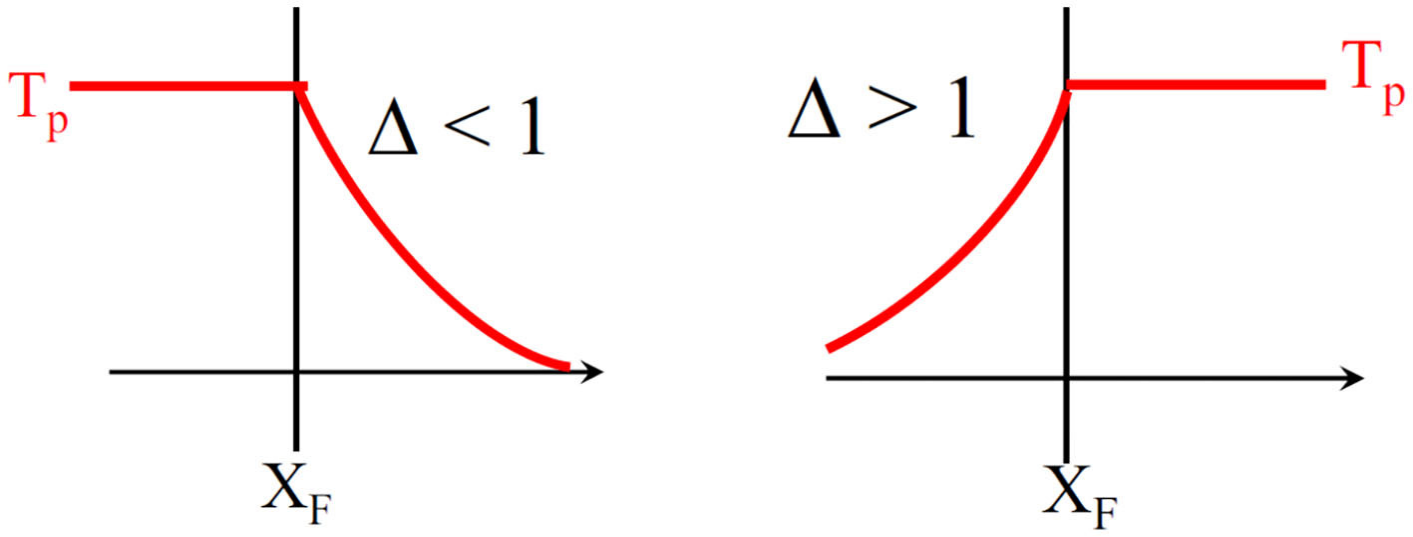
Analytical solutions for different regimes depending on Δ values.

The solution of Equation (31) is written in Table 3. The solutions are summarized in Figure 5 and the plateau temperature is defined by

(34) $$\begin{array}{l} T_{p}=\frac{T_{ad}}{\left|△-1\right|}. \end{array}$$

If △ < 1, the heat remains stored before the reaction front, this regime is called reaction leading structure. If △ > 1, the heat is transferred by convection in front of the reaction region. The last case corresponds to △ = 1. In this case, all the heat remains located in the front. The front is moving at the same velocity as the heat. There is no stationary solution. It is important to note that if 0 < △ < 2, *T*_*p*_ will be higher than *T*_*ad*_. This is called the superadiabatic effect, reported in many studies (Aldushin et al., 1999; Wahle and Matkowsky, 2001). Parameters △, *T*_*p*_ and *T*_*ad*_ are used in our simulations in order to characterize the regimes but also as indicators for the validation of our model.

**Table 3:** Solutions of equation.

	***x* < *x*_*F*_**	***x* > *x*_*F*_**
△ > 1	*T*(*x*) = *T*_*p*_exp(*x*/*L*)	*T*(*x*) = *T*_*p*_
△ < 1	*T*(*x*) = *T*_*p*_	*T*(*x*) = *T*_*p*_exp(−*x*/*L*)

### 2.4. Numerical Methods

The model is implemented in COMSOL Multiphysics toolbox. We develop a fully coupled solver rather than a sequential approach. The convergence criteria are set so that the residuals of all equations are less than 10^−5^. Furthermore, a sensitivity analysis was conducted in order to guarantee that all of the results in the present study are independent of the grid size.

## 3. Results and Discussion

In the first step, validation tests are done, in the same way as Yang and Debenest (2014). According to the imposed values of concentrations and flow rate, we can estimate the expected values of front velocity and plateau temperature. All these tests are done using the 1D geometry (see Appendix 1). In the second step, we will investigate in the same way as Baud et al. (2015), some of the information like front width and disequilibrium between the fluid and particle temperatures, by varying the carbon content or flow rate. This will be done using the 2D symmetric media. The last subsection is devoted to more complex geometries for which different flow regions could exist. This will allow to address some possible effects of heterogeneities in the case of a complex porous medium.

### 3.1. Validation Cases

In Appendix 1, we report the results for a simplified 1D geometry, on which Darcy's law is firstly used rather than the full DBM. In this case, all of the transport equations used only concern the porous media (Equations 5 and 20). But, the simulations are done using a full compositional and dilatable formulation. We refer the readers to the Appendix in order to verify the validity of the porous medium formulation and its validation compared to analytical predictions. To validate the complete DBM numerical model, the composite symmetric medium, illustrated in Figure 3, is used. We will first investigate the effect of Δ variation, in a range where previous results are available. Then, *Pe*_*p*_ will be varied in order to understand its effect on the reaction front length.

#### 3.1.1. Influence of Δ

Three different mass percentages of carbon close to the values of Baud et al. (2015) are used in this validation step. Values range from 2 to 4% of carbon mass fraction, assuming that pure carbon is deposited in the medium, inside the grains. All the other parameters come also from Baud et al. (2015). As reported in Table 4, a good agreement between predicted and analytical values can be observed. We have reported *Tp* (*K*) and *U*_*F*_ comparing these values to the analytical predictions given by the simplified analytical problem. This is exactly the same procedure than the one in Yang and Debenest (2014).

**Table 4:** Comparison between expected analytical and numerical values for the symmetric medium.

	**% C**	***Pe*_*p*,*O*__2_**	**Δ_*th*_**	***T*_*p,th*_ (K)**	***U*_*F,th*_ (m/s)**	**Tp **(*K*)****	***U*_*F*_ (*m*/*s*)**
Sim. #1	2	2.5	0.18	1063	1.87 × 10^−4^	1063	1.87 × 10^−4^
Sim. #2	3	2.5	0.27	1602	1.244 × 10^−4^	1602	1.25 × 10^−4^
Sim. #3	4	2.5	0.36	2273	8.8 × 10^−5^	2272	8.8 × 10^−5^

*Note that T_p,th_ refers to Equation (34) and U_F ,th_ to Equation (26)*.

In the following, the fields are normalized as

– *Y*_*adim*_ = *Y*_*f*_ ,_*O*_2__/*Y*_*f*_ ,_*O*_2,*in*__ for the normalized mass fraction of oxygen;
– *T*_*adim*_ = (*T*−*T*_*ref*_)/*T*_*ad*_ for the normalized temperature field.

The colorbar is then the same for all these normalized visualizations, and range from dark blue for 0 to dark red for 1. For the normalized temperature the superior limit is set to 1.2 because the temperature could exceed the adiabatic one.

**Figure 6:**
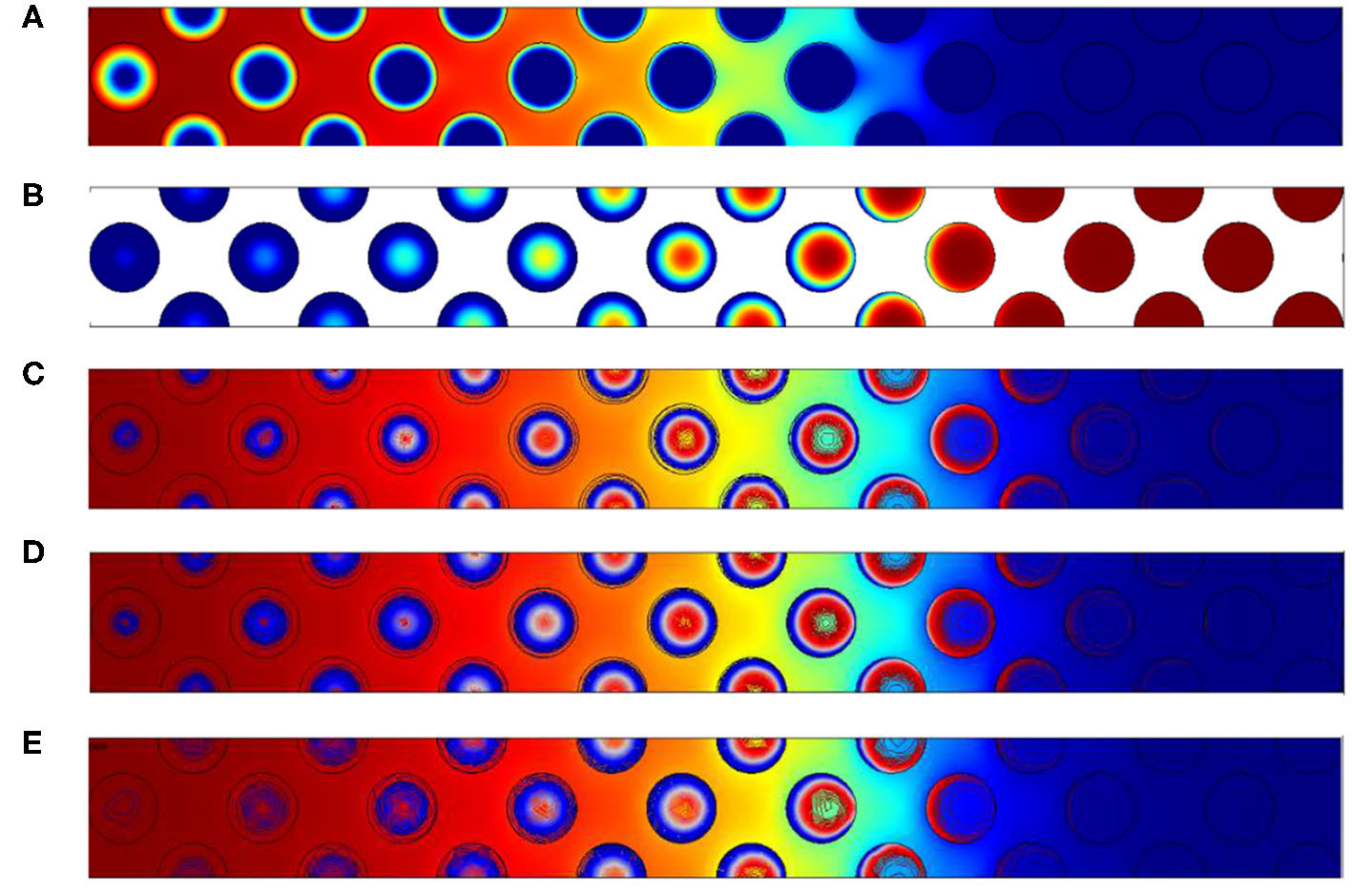
Contours of **(A)** normalized mass fraction of oxygen **(B)** normalized carbon content and **(C)** normalized temperature for the configuration with 2% of carbon. Normalized temperatures for the other configuration with **(D)** 3% of carbon and **(E)** 4% of carbon. The Péclet value imposed is always *Pe*_*p*,_*O*__2__ = 2.5.

Figure 6 illustrates oxygen, carbon and temperatures numerically observed in the vicinity of the front. As the activation energy is low, the length of the reaction zone is only governed by the amount of oxygen. This is imposed by the oxidant Péclet number which is constant in this situation. So as Δ varies, the only expected differences are related to temperature fields.

Theses illustrations also show that the reaction occurs within the grains, and oxygen is depleted firstly on the grain surface. Oxygen then enters in the grains as it is confirmed by the carbon concentration field (Figure 6B). On the left side, i.e., where the oxygen feeding arrives, a uniform concentration is observed in the pores and decreases inside the grain. In the reaction region, the oxygen concentration decreases in a circular form, directly related to the presence of the carbon content. The front's length is relatively important, close to 10 grain diameters. The concentration contours are almost symmetric inside the grains. It is a consequence of the oxygen Péclet value which is higher that unity in the free region and ensure a uniform concentration on all the grain surface. Increasing Δ results in the rise of temperature. As expected, in the reaction zone, values are always higher than 1. The superadiabatic effect is clearly recovered as expected by the analytical prediction.

#### 3.1.2. Variations of *Pe*_*p*_

In this studied configuration, the carbon content is fixed to 3% and inlet velocities vary from 0.021 to 0.168 m/s, similar to Baud et al. (2015) whose values were ranged between 0.021 and 0.210 m/s). The chosen inlet velocities correspond to *Pe*_*p*,_*O*__2__ = 2.5, 10, 20.

**Figure 7:**
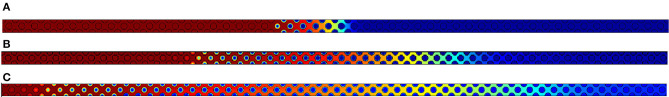
Oxygen concentration fields obtained respectively for **(A)** *Pe*_*p*,_*O*__2__ = 2.5, **(B)** *Pe*_*p*,_*O*__2__ = 10 and **(C)** *Pe*_*p*,_*O*__2__ = 20. Concentration varies from 0 in dark blue to its maximum value 1 in dark red.

As expected, Figure 7 permits to observe that increasing the Péclet number leads to enlarge the reaction region. Two supplementary effects could also be expected when increasing Péclet values:

– The first is an escape of oxygen at the outlet of the computational domain and then a variation of Δ, as mentioned in Debenest et al. (2008);
– The second is the enhancement of local thermal disequilibrium which is directly related to the thermal Péclet number *Pe*_*F,s*_.

These effects are not observed here because the temperatures are very high in the vicinity of the reaction zone. According to available works (Debenest et al., 2005b, 2008), when this Péclet number is larger than unity, large thermal disequilibrium could exist, within the solid phase or between each phase. In our case, the front velocity *U*_*F*_ remains smaller than 10^−4^ m/s, and *Pe*_*F,s*_ is always smaller than unity. In Debenest et al. (2005b), the thermal equilibrium Péclet number, has been defined. It compares the time scales based on the front velocity to the time scales for the heat conduction within the solid. The local thermal equilibrium is observed, within and between the different phases presented in this model.

As, we only focus on the comparison with experimental data in the next part, we will not give any other indications about this analysis, reporting the readers to previous studies.

### 3.2. Synthetic Dual-Porosity Media

We focus on another configuration (Figure 4) which present preferential pathways as in the experimental case (Figure 1). We first vary the Péclet number *Pe*_*p*,_*O*__2__ = 0.625, 2.5, 25 by changing the inlet velocity. The carbon concentration is fixed, equal to 3% in mass. It is initially deposited uniformly inside the grains. A constant inlet temperature of 650 K is imposed to ignite the bed.

To make figures comparable in term of injected quantity of oxygen, we also define a non-dimensional time *t*′

(35) $$\begin{array}{l} t^{\prime }=\frac{U_{in}.t}{L_{ref}}, \end{array}$$

which represents an adimensional time with respect to the characteristic convection time *U*_*in*_/*L*_*ref*_.

**Figure 8:**
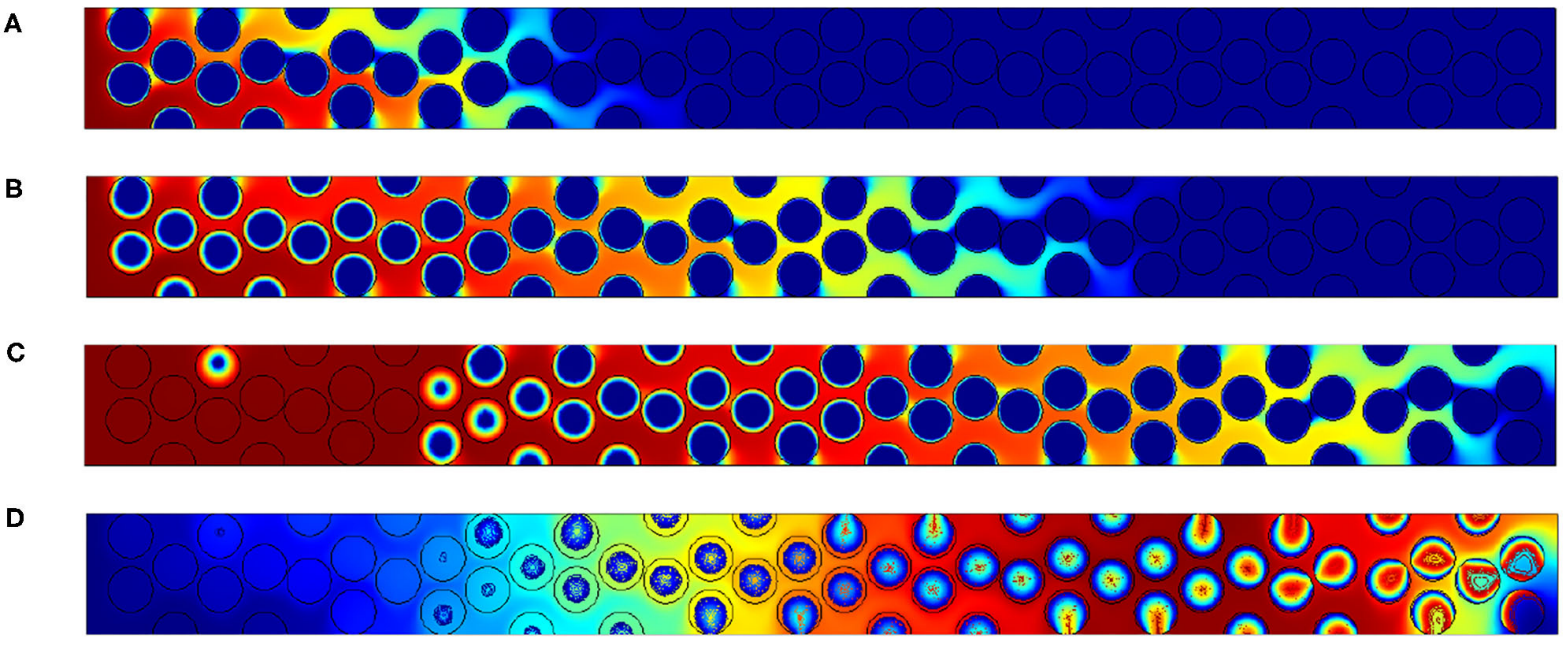
Results for *Pe*_*p*,_*O*__2__ = 25. Oxygen concentration contours at **(A)** *t*′ = 6, 000, **(B)** *t*′ = 18, 000 and **(C)** *t*′ = 30, 000. **(D)** Field of temperature and contours of carbon content within the grain at *t*′ = 30, 000.

For the largest value of *Pe*_*p*_,_*O*_2__ = 25 illustrated in Figure 8, the reaction region is extended up to 20 diameters. Between the time *t*′ = 9, 000 and *t*′ = 15, 000, respectively, Figures 8B,C, oxygen reaches the outlet without reacting with the porous medium. This is mainly due to the intragranular diffusion which limits the reaction rate. This will decrease the front velocity and thus increase the value of Δ. This was also experimentally observed in Baud et al. (2015) where half of the oxygen cross the bed without reacting with the carbon content.

**Figure 9:**
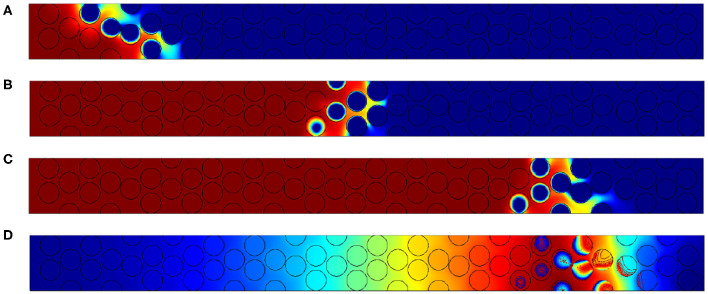
Results for *Pe*_*p*_ = 2.5. Oxygen concentration contours at **(A)** *t*′ = 6, 000, **(B)** *t*′ = 18, 000, and **(C)** *t*′ = 30, 000. **(D)** Field of temperatures and contours of carbon content within the grain at *t*′ = 30, 000.

**Figure 10:**
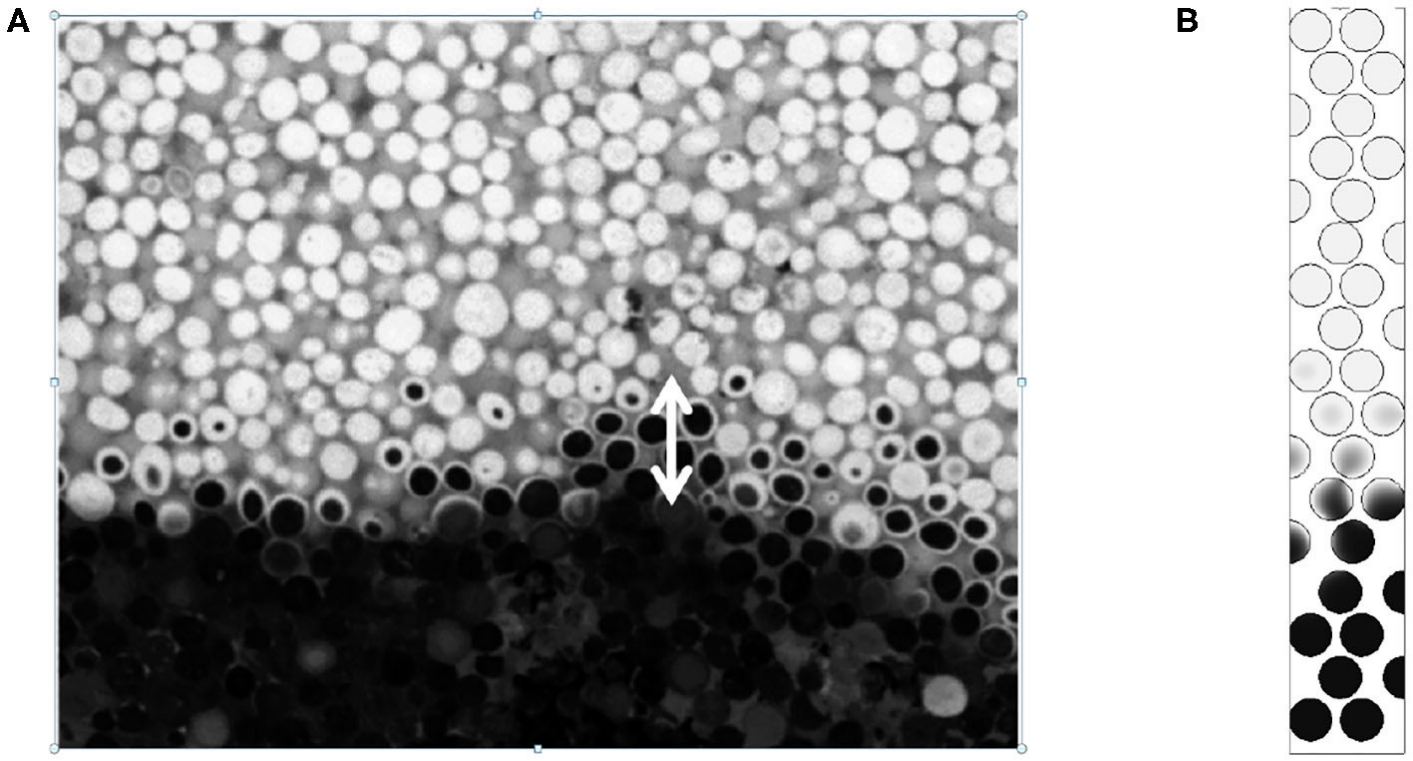
Carbon content in the front region obtained for the same thermochemical conditions as Baud et al. (2015): *Pe*_*p*_ = 2.5 and %C = 3. **(A)** Experimental visualization and **(B)** normalized carbon content obtained from numerical simulation. For the numerical simulation the normalized carbon content is colored from 0 (black) to 1 (white). Reprinted (adapted) with permission from Baud et al. (2015). Copyright (2015) American Chemical Society.

In the case where *Pe*_*p*_ = 2.5 (Figure 9) the reaction region is reduced to 4 or 5 diameters. We can compare with the available experimental data reported in Figure 10 obtained by cutting the combustion zone post mortem. One can visualize using the gray scale the carbon rich and carbon poor zones inside the particle. The chemical front thickness can be defined as the distance between virgin particles (in black) and totally oxidized particles (white).

In Figure 10, the width of the reaction zone obtained by numerical simulations is close to the zone observed experimentally. Moreover, the carbon concentration contours given both from experiments and numerical simulations exhibit the same decay, with comparable patterns, the concentration decreasing on the crown rather than in volume.

**Figure 11:**
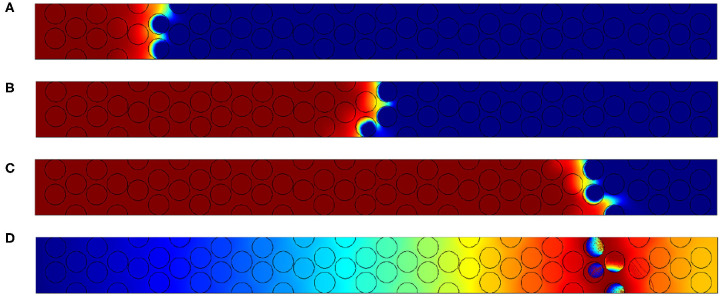
Results for *Pe*_*p*_ = 0.625. Oxygen concentration contours at **(A)** *t*′ = 6, 000, **(B)** *t*′ = 18, 000, and **(C)** *t*′ = 30, 000. **(D)** Field of temperatures and contours of carbon content within the grain at *t*′ = 30, 000.

For the lowest Péclet number illustrated in Figure 11, the characteristic length of the reactive zone is close to one diameter. A temperature field completed with the carbon content (Figure 11D), highlights the uniformity of the temperature field with no strong effects of thermal non-equilibrium and a sharp front for the carbon consumption.

**Figure 12:**
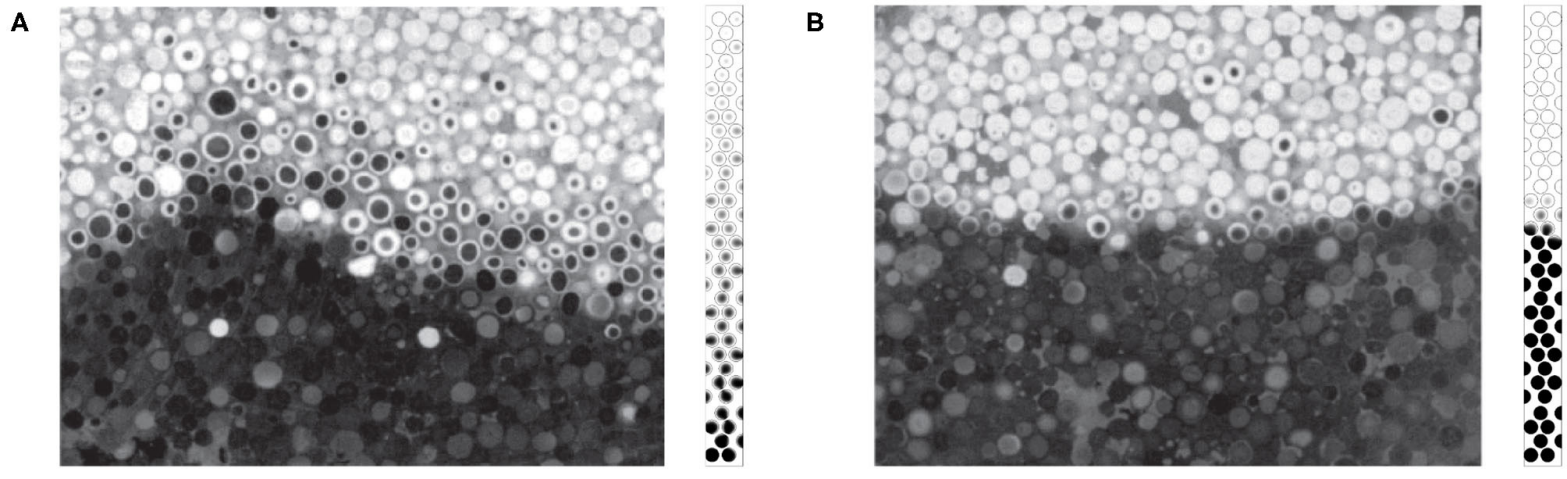
Comparisons between experiments and numerical simulations (Carbon content 2.3 %) for **(A)** *Pe*_*p*_ = 25 and **(B)** *Pe*_*p*_ = 2.5 (for the color, same convention as Figure 10). Reprinted (adapted) with permission from Baud et al. (2015). Copyright (2015) American Chemical Society.

Simulations are also performed by reducing the carbon content to 2.3% of the mass. This allows to compare numerical results with experimental results obtained by Baud et al. (2015) in this configuration, for *Pe*_*p*_ = 25 and *Pe*_*p*_ = 2.5. In Figure 12, we observe comparable results. The width of the reactive front is close to 1 particle diameter when Pe tends to 1 while it increases to 5–10 particle diameters for larger Péclet values.

In Figure 12, we clearly see the spreading of the front with the decreasing amount of carbon and the increasing Péclet number. It is difficult to compare directly, but a size close to 15 diameters seems to be representative of the experimental cuts when *Pe*_*p*_ = 25. In the numerical counterpart, the spread seems larger compared with available experimental data. But, on the left part, some localized grains are not burnt, increasing the front width to more than 20 diameters. The boundary conditions are possible explanations (temperature equal to 650K at the inlet), but also the length of the numerical domain, too small to establish the front. This effect is also visible on the righten part of Figure 12. Even if the front is sharp, we clearly detect some localized unburnt particles behind the front. Numerically, no thermal losses are imposed on the lateral sides, and this could explain partly the presence of this localized unburnt grains.

In the next section, we use the simulations done at *Pe*_*p*_ = 2.5 and 25 in order to investigate the effects of the inner grain permeability values on the model.

### 3.3. Influence of the Inner Particles Permeability

Due to uncertainties in the values of the inner particle permeability, we have chosen to make it vary along a possible range. Thanks to available experimental data and SEM images, the pore size has been determined to be close to 40 nm in the particles. Thus we can expect the permeability of the particles to be small, may be less than 10^−14^ m^2^, the value set for all previous calculations. Anyway, those microporous particles can conduct the flow, and that is why we have decided to use a DBM approach rather than a classical Navier Stokes formulation of the flow, ignoring the possibility for the flow to convey heat and mass within the particles. In order to test the sensitivity of the results to this parameter, we choose five values of permeability namely, 10^−14^ m^2^, 10^−12^ m^2^, 10^−10^ m^2^, 10^−9^ m^2^, and 10^−8^ m^2^ for a Péclet value *Pe*_*p*_ = 2.5 and a carbon content of 3% in mass. The adimensional time *t*′ is taken equal to 8,400.

**Figure 13:**
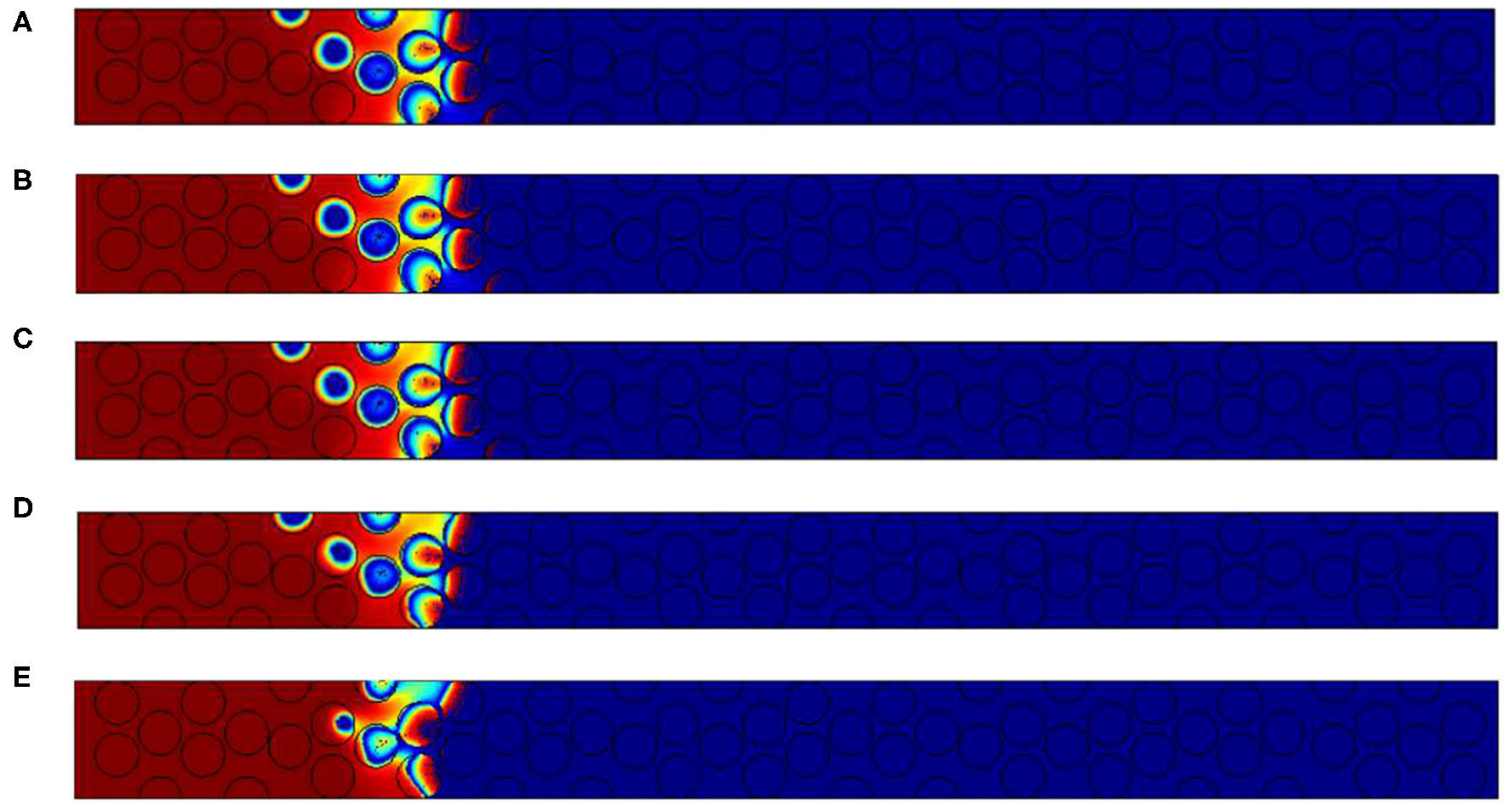
Results for *Pe*_*p*_ = 2.5. Oxygen concentration contours and carbon content for different inner grain permeability K, **(A)** 10^−14^ m^2^, **(B)** 10^−12^ m^2^, **(C)** 10^−10^ m^2^; **(D)** 10^−9^ m^2^ and **(E)** 10^−8^ m^2^ (for the color, same convention as Figure 10).

The major effect of the inner grain permeability increase is the shortening of the reaction zone. From the top image to the bottom one of the Figure 13, the size of the reaction region is close to 5 particles when the permeability value are in less than *K* = 10^−9^ m^2^. No important differences exist in this case between cases (a) to (d). But when 10^−8^ m^2^, the reaction length is reduced to 3 particle diameters. In order to accurately check this effect we will increase the Péclet values for *Pe*_*p*_ = 25.

This is also visible when increasing the Péclet values for *Pe*_*p*_ = 25. For this figure, the permeability values chosen are 10^−14^ m^2^ and 10^−8^ m^2^ and the adimensional time *t*′ is equal to 27,600. We represent there both the contours of oxygen and carbon contents (a and b), but the velocity in the particles is also given using the logarithmic function *log*(*U*/*U*_*in*_) in the scale varying from −15 to 1 (c and d).

**Figure 14:**
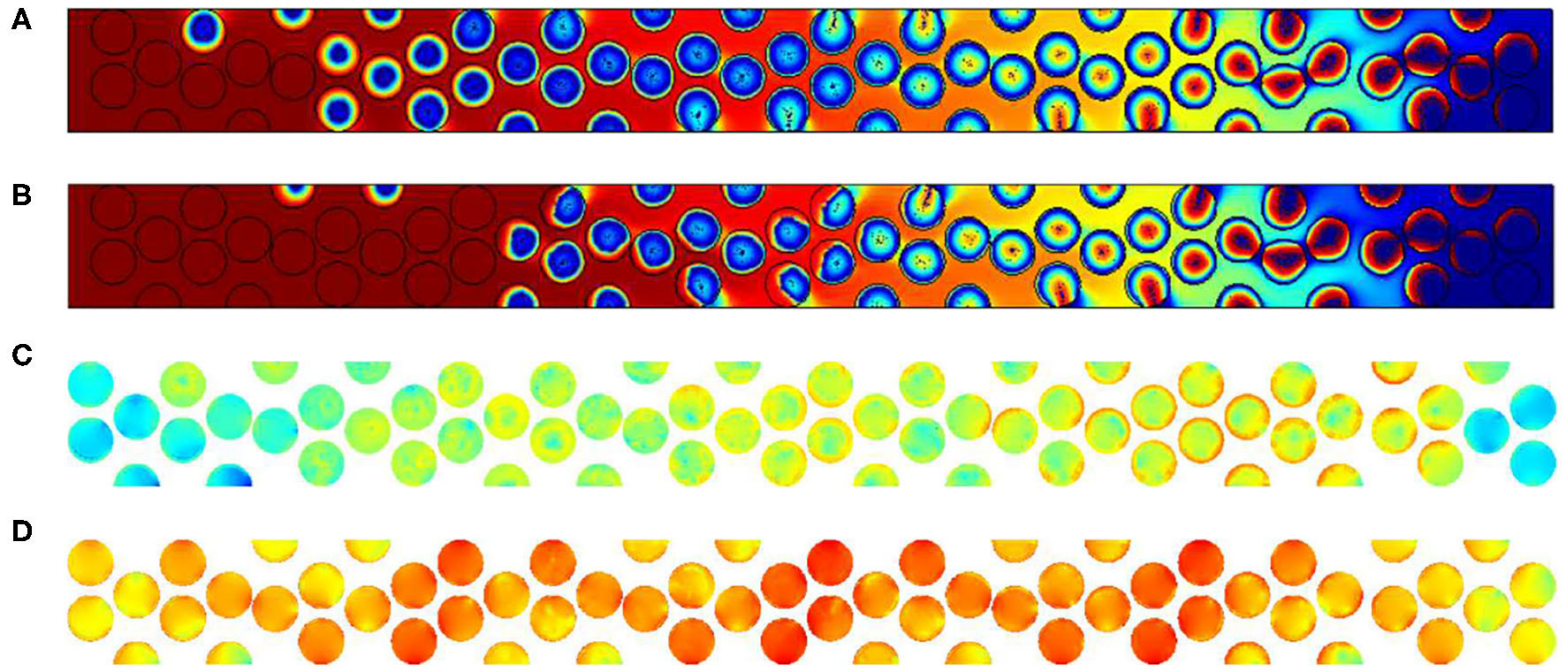
Results for *Pe*_*p*_ = 25. Oxygen concentration contours and carbon content for different inner grain permeability K **(A)** 10^−14^ m^2^, **(B)** 10^−8^ m^2^, **(C)** *log*(*U*/*U*_*in*_) for 10^−14^ m^2^, and **(D)** *log*(*U*/*U*_*in*_) for 10^−8^ m^2^ (for the color, same convention as Figure 10).

Comparing Figures 14A, B corresponding to simulations performed for K equal to 10^−14^ m^2^ and 10^−8^ m^2^, we observe that the contours of oxygen and carbon contents are decaying in a shorter range. It is due to the importance of the flow within the grains. This flow is enhanced as we decrease the viscous resistance inside the particles. To check this assumption, we plot in Figures 14C, D the velocity field normalized by the inlet velocity in log only in particles. The color code is once again the same, varying from minimum values in dark blue (10^−15^) to maximal values (1) in red. Figure 14C exhibits a small impact of the flow when permeability is low. Increasing the inner particle velocity eases the flow within the particles, conveying oxygen to the available reactive material. According to the previous experimental results discussed in the previous section, we can expect that the values of inner grains permeability are more important than 10^−8^ m^2^, which corresponds to a porous medium with micropores dimensions close to 0.1 to 1 micrometer.

## 4. Conclusion

In the present study, we develop a Darcy-Brinkman model to carry out simulations of the smoldering process in simplified porous medium geometries. After validating the multicomponent and compressible model comparing the results to analytical solutions, in the same way than Yang and Debenest (2014), we study the effects of flow rate and reactive material composition variations in a 2D symmetric geometry. Then, we focus on a more detailed image of what could be a porous medium and try to compare with imposed carbon contents and flow rates from Baud et al. (2015).

We use these recent available experimental data focusing on front width obtained by longitudinal cuts. The results from the Darcy-Brinkman model is compared favorably in different situations, i.e., carbon contents and for different mass flow rates. So, this model is able to capture some of the main features of the physical problem even if strong assumptions are present (no radiative heat transfers, no variations of properties with the reactive material concentration, local thermal equilibrium in the particles).

A sensitivity analysis to the iner grain permeability has been done, demonstrating that the front length is reduced when the inner grain flow becomes significant.

In a forthcoming study, the variations of inner grain properties will be taken into account into the effective properties used. The Darcy-Brinkman formulation allows the porosity and permeability values to evolve, with the fuel content for instance, during the reactive process and this will be used to treat the special case of burning particles. Assuming that the inner grain porosity increases when the fuel is consumed, and relating the porosity to the permeability will allow to treat the disappearance by fuel consumption of the particles.

This work makes it possible to validate the DBM approach and to envisage the development of a dedicated three-dimensional tool able to treat more complex situations.

## Data Availability Statement

The raw data supporting the conclusions of this article will be made available by the authors, without undue reservation, to any qualified researcher.

## Author Contributions

GD wrote most of the paper and perform 2D simulations. PH and RG have written the mathematical model and perform part of the 1D validation test cases. CY have polished and written partly of the results part. All authors contributed to the article and approved the submitted version.

## Conflict of Interest

The authors declare that the research was conducted in the absence of any commercial or financial relationships that could be construed as a potential conflict of interest.

## Appendix: 1D Darcy-Scale Medium

For the 1D geometry, the operating parameters used in the simulations are reported in Table A1. All the other material properties are the ones defined previously. We decide to change only the carbon content and then modify the front velocity and the temperature levels. It is also important to point out that modifying the Péclet number will not change the regime in terms of values of (△, *Pe*_*O*_2__). For each simulation, we provide in Table A1, the values of the front velocity and the temperature levels compared with the one expected from the analytical predictions.

**Table A1:** Parameters used for the 1D validation tests.

**Parameters**	**ρ_*c*_ (*kg*/*m*^3^)**	**△*H*_*r*_ (*J*/*Kg*)**	***U*_*in*_ (m/s)**	***Y*_*O*__2_**	**△**	***T*_*ad*_ (K)**	***Pe*_*p*_,_*O*__2_**
Sim. #1	7.3875	2.569 × 10^6^	0.02	0.02	0.125	287	2.62
Sim. #2	14.75	2.569 × 10^6^	0.02	0.02	0.25	57	2.62
Sim. #3	18.4375	2.569 × 10^6^	0.02	0.02	0.312	115	2.62
Sim. #4	29.5	2.569 × 10^6^	0.02	0.02	0.5	230	2.62
Sim. #5	295	2.569 × 10^6^	0.02	0.02	5	1098	2.62
Sim. #6	590	2.569 × 10^6^	0.02	0.02	10	228	2.62

Table 4 shows that the relative error (between theoretical and numerical values) does not exceed 0.4%. This demonstrates that the stoechiometry is respected and validates the coupling between mass transport phenomena and reactions of the solid residue. In the case of temperature levels, the agreement is once again very good for low values of Δ. For higher values, the relative error is maximal for Δ = 5 and close to 2%. This is possibly due to the slow establishment of the solution and the influence of the outlet boundary conditions where a convective flux condition is imposed with a thermal Péclet number close to unity. The case Δ = 5 is the most sensitive case as the thermal wave is longer to establish (larger value of Plateau temperature than in the case of Δ = 10). Even if we could investigate the case of a longer porous medium to deal with this technical issue, we consider that the Darcy combustion model is valid.

**Table A2:** Comparisons between analytical predictions and numerical simulations (1D test-case).

**Parameters**	**△**	***V*_*f*_ (m/s) (Equation 26)**	***V*_*f*_ (m/s) num**.	***T*_*p*_ (K) (Equation 34)**	***T*_*p*_ (K) num**.
Sim. #1	0.125	2.97 × 10^−4^	2.97 × 10^−4^	324	324
Sim. #2	0.25	1.48 × 10^−4^	1.48 × 10^−4^	366	366
Sim. #3	0.312	1.186 × 10^−4^	1.19 × 10^−5^	392	391
Sim. #4	0.5	7.43 × 10^−5^	7.40 × 10^−5^	512	511
Sim. #5	5	7.43 × 10^−6^	7.32 × 10^−5^	567	556
Sim. #6	10	3.71 × 10^−6^	3.7 × 10^−6^	532	526
